# Artificial intelligence as the new frontier in chemical risk assessment

**DOI:** 10.3389/frai.2023.1269932

**Published:** 2023-10-17

**Authors:** Thomas Hartung

**Affiliations:** ^1^Center for Alternatives to Animal Testing (CAAT), Bloomberg School of Public Health and Whiting School of Engineering, Johns Hopkins University, Baltimore, MD, United States; ^2^CAAT-Europe, University of Konstanz, Konstanz, Germany

**Keywords:** computational toxicology, machine learning, big data, regulatory toxicology, scientific revolution

## Abstract

The rapid progress of AI impacts various areas of life, including toxicology, and promises a major role for AI in future risk assessments. Toxicology has shifted from a purely empirical science focused on observing chemical exposure outcomes to a data-rich field ripe for AI integration. AI methods are well-suited to handling and integrating large, diverse data volumes - a key challenge in modern toxicology. Additionally, AI enables Predictive Toxicology, as demonstrated by the automated read-across tool RASAR that achieved 87% balanced accuracy across nine OECD tests and 190,000 chemicals, outperforming animal test reproducibility. AI’s ability to handle big data and provide probabilistic outputs facilitates probabilistic risk assessment. Rather than just replicating human skills at larger scales, AI should be viewed as a transformative technology. Despite potential challenges, like model black-boxing and dataset biases, explainable AI (xAI) is emerging to address these issues.

## Introduction

At the 2022 International Conference of Toxicology, the traditional EuroTox and SOT debate addressed the question “Is there a role for AI and machine learning in risk decisions?.” Representing EuroTox, I argued for AI’s role, while my esteemed colleague Craig Rowlands argued against for SOT, with our stances reversed from earlier that year at SOT in San Diego. However, the debate question should have been “Is there a risk assessment role without AI?.” AI permeates all of science, so why should toxicology be any different? In 2021, 2.5% of scientific publications already included AI, with Stanford’s Artificial Intelligence Index Report estimating total AI publications at half a million. The AI industry is expanding rapidly with a 38% CAGR (Compound Annual Growth Rate).

AI capability gains are most striking: In March 2022, GPT-4 was released. On simulated bar and SAT exams, GPT-4 performed at the 90th and 93rd percentiles, respectively. Its 89th percentile SAT Math performance is notable given these exams reflect US high school education. While we may critique lawyers and high schools, such performance remains impressive, especially compared to GPT-3.5 one year prior at the 10th percentile. Could risk assessment be more intellectually taxing?

AI, a.k.a. machine learning, has become exceptionally disruptive due to synergies between computational power growth, the accumulation of “big” data, and machine learning optimization. To quantify this disruption, computer power doubles every two years as per Moore’s law (named after Intel’s Gordon Moore), with costs halving. Global data is estimated to grow ~60% annually - over 90% of all data was generated in the past four years. Since achieving human-level performance on certain 2012 pattern recognition tasks, AI capability has doubled every three months. This is exemplified by GPT-4, trained on only ~1 million times more parameters than GPT-3 yet dramatically outperforming it two years later. Together, AI power has increased over one billion-fold in the past 50 years.

These technological strides impact all aspects of life, including toxicology (Santín et al., 2021; Jeong and Choi, 2022; Lin and Chou, 2022; Tran et al., 2023). Obvious examples are information retrieval and data extraction. Computers reading scientific papers with student-level comprehension are imminent, except the computers will be able to process millions of papers simultaneously without forgetting any information. While tables and figures pose current challenges, these will be overcome in time. Digital pathology with image analysis and sharing will also affect toxicology, as will big toxicology data from increasingly curated legacy studies, open machine-readable literature, internet grey literature (e.g., >900,000 safety data sheets), sensor technologies, high-throughput testing (ToxCast, Tox21), and omics platforms. We have progressed far from simply counting dead animals.

Modern toxicology’s key challenge is integrating these multifarious information sources, a task uniquely suited to AI and machine learning. In 2018, we demonstrated RASAR (read-across-based structure activity relationships), an automated read-across tool, achieving 87% balanced accuracy across nine OECD tests and 190,000 chemicals in five-fold cross-validation. In the same study, six OECD animal tests averaged 81% reproducibility (Luechtefeld et al., 2018). Accuracy improved through transfer learning *via* input of 74 chemical properties, fusing diverse data. While humans can handle a few parameters, AI excels with big data - characterized by volume, variety, and velocity. AI data analysis outputs probabilities, enabling probabilistic risk assessment (Maertens et al., 2022). The EU ONTOX project explores AI opportunities from a natural language processing perspective for data extraction to enable predictive toxicology *via* probabilistic risk assessment (Vinken et al., 2021). Recently, we compiled a database of 200 million chemical/property/result triplets as a computational toxicology resource.

However, AI should not just replicate human skills at scale. In 2022, DeepMind’s AlphaZero matched world chess champion Magnus Carlsen’s 2,882 Elo rating after ~4 h of self-play, reaching an unbeatable 3,581 Elo after 8 h. Remarkably, AlphaZero played chess unconventionally, leading professionals to study its novel strategies. We should expect AI not only to perform our work, but to find new ways to do so.

In summary, AI brings both challenges and promise for toxicology. Black-box aspects of many methods may impede regulatory acceptance, although explainable AI (xAI) is advancing and may catch up. Data biases are concerning, but if humans can identify them, machines can too. While humans must oversee AI, it provides readily condensed information. User interfaces are also improving continuously. However, AI literacy among students and regulatory/industry professionals raises concerns. Toxicology degrees must include AI training, and we must embrace “ToxAIcology,” as it is coming regardless.

### AI fundamentals for toxicology

Chinese AI specialist Fei-Fei Li noted “I often tell students not to be misled by ‘artificial intelligence’ - there is nothing artificial about it. AI is made by humans, intended to behave by humans, and, ultimately, to impact human lives and society.” Though often used interchangeably, artificial intelligence (AI) and machine learning (ML) have distinct definitions:

- AI refers broadly to mimicking human intelligence *via* machines/software, with the goal of performing tasks requiring intelligence. AI types include narrow AI, designed for specific tasks like voice commands, and general AI, capable of any intellectual human task.
- ML is an AI subset and data analysis method enabling algorithm improvement without explicit programming. ML models learn from data and enhance accuracy over time, especially deep learning models, as exposure to data grows. ML categories include supervised, unsupervised, and reinforcement learning.

In summary, AI is the broad field encompassing approaches to machine intelligence, while ML specifically involves data-driven algorithm improvement. As the most practical/successful AI form currently, ML is often used interchangeably with AI.

### Other key concepts

- Natural Language Processing (NLP) focuses on human-computer interaction through natural language to understand and extract value from language.
- Reinforcement Learning involves agents maximizing rewards by taking actions in an environment. It is critical for training AI systems dynamically.
- Neural Networks are algorithms designed to recognize data patterns *via* machine perception, labeling, and clustering.
- Expert Systems emulate human expert decision-making to solve complex problems *via* reasoning and knowledge.

These types and subfields overlap, with many AI systems combining multiple techniques.

## AI trends and promise for toxicology

AI has expanded rapidly and will continue doing so. Increasing AI integration into daily life is expected, from personalized media recommendations to voice assistants. This trend should persist, with AI becoming more pervasive and seamless. AI holds vast healthcare potential, from disease prediction *via* big data to personalized treatments, new drug development, and care delivery improvements – as technologies mature and regulations evolve. Several trends will shape AI’s future, relevant to toxicology:

- AI Ethics and Regulation: As AI permeates society, ethical and regulatory considerations will become critical regarding data privacy, algorithmic bias, automation impacts, etc. Consumer and patient safety concerns magnify this importance in toxicology.
- FAIR Data: Quality data is the basis of AI, requiring findability, accessibility, interoperability, and reusability. Traditionally, quality assurance in computational toxicology was achieved by data curation, but Big Data pose enormous challenge to any manual curation. However, ultimately, what humans can curate, machines can as well. Increasingly, we will have to employ AI itself to identify biases, quality-assure and curate datasets.
- Synthetic Data: This artificially created data retains the properties and business value of the original data while ensuring privacy compliance. Toxicology could explore its use for data sharing without compromising commercial interests.
- Explainable AI (xAI): Many AI models are “black boxes,” limiting trustworthiness and transparency. xAI aims to provide understandable explanations to increase model interpretability and acceptability, especially for regulatory purposes.
- Generative AI: Generative AI can produce text, images, and other media in response to prompts by learning patterns and structure from training data. Reporting, documentation, and communication could benefit in toxicology.
- Federated Learning: This distributed machine learning approach trains algorithms on multiple decentralized datasets. For example, a predictive toxicology tool could train behind company firewalls without sharing data.
- Automated Machine Learning (AutoML): AutoML automates applying machine learning to real-world problems, making it more accessible to non-experts while improving expert efficiency. New toxicology insights could rapidly inform stakeholders.
- Quantum Computing: Quantum computers will significantly increase computing power once realized. This could enable solving more complex problems in systems biology/toxicology and complex modeling.
- Data-Centric AI: This AI approach focuses on data operations like cleaning and labeling rather than model parameters. It will be important for curating toxicology legacy data.

### Expert systems – democratizing toxicology expertise

Expert systems are AI programs using reasoning and knowledge to solve problems, providing expert advice for medicine, engineering, etc. They comprise a knowledge base and inference engine applying rules/logic to solve problems. Though not infallible, expert systems enhance decision-making quality.

Advantages include providing otherwise inaccessible expertise, solving difficult/time-consuming problems, and improving decision consistency/accuracy. Disadvantages include development/maintenance costs, updating difficulties, and potential knowledge base biases.

Early toxicology expert systems include DEREK (toxicity prediction), METAPC (biodegradation), METEOR (metabolic predictions), OncoLogic (carcinogenicity prediction), and StAR. As chatbots expand toxicology knowledge access, expert systems may resurge with challenges/opportunities, especially for lay audiences.

### Future AI trends in toxicology

AI can revolutionize toxicology *via* improved toxicity understanding, risk prediction, and treatment development. AI is already enhancing prediction *via* big data, identifying at-risk individuals, and informing interventions. Other expected innovations include:

AI-Enhanced Toxicity Understanding: AI will better elucidate chemical toxicity by finding patterns in large datasets like HTS and epidemiological data to develop new safety regulations and safer chemical design.

Improved Risk Prediction: AI will increasingly forecast chemical exposure toxicity risks for individuals. This facilitates interventions to reduce high-risk individuals’ exposures.

Novel Treatment Development: AI can identify new poison treatments and diagnostics and optimize care delivery to poisoned patients. It may also enable personalized toxicology *via* genetic/epigenetic data.

However, realizing these benefits requires addressing:

Data Availability/Quality: AI depends on high-quality, well-curated, unbiased, and representative training data. Toxicological databases meeting FAIR principles are essential.

Animal Testing Ethics: While reducing animal testing, AI efficiency gains could also enable more substances to be tested. New ethical questions arise.

Interpretability: Complex AI models like deep learning are poorly understood “black boxes,” limiting mechanistic insights - critical in toxicology.

Overall, AI presents exciting opportunities through enhanced prediction, reduced animal testing, and other innovations. Carefully addressing these challenges can lead to transformative contributions of AI to advancing toxicology and safety.

## Conclusions on AI’s emerging toxicology role

**Figure 1:**
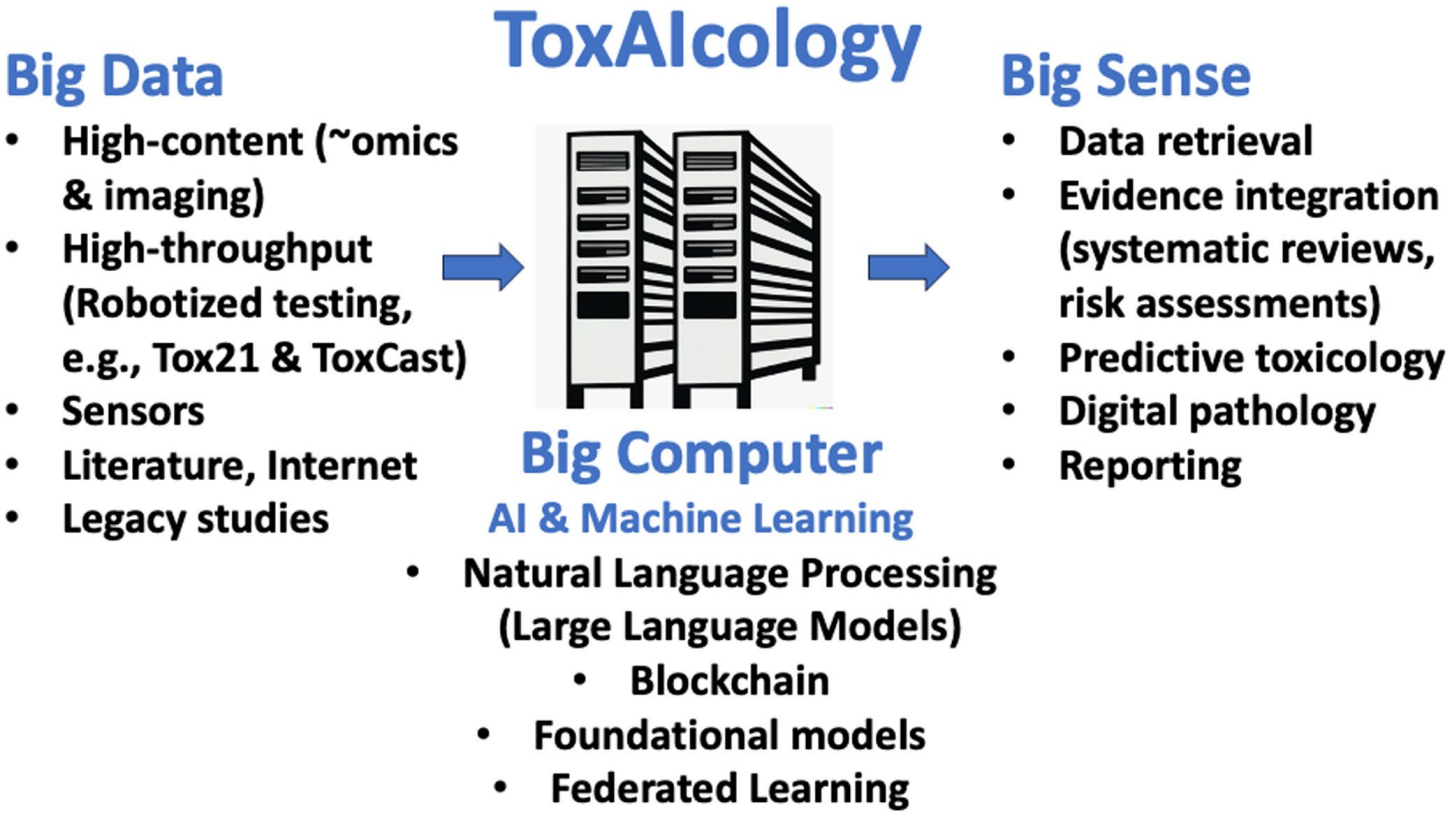
ToxAIcology - AI-assisted toxicology. The figure depicts how new approaches are increasingly providing Big Data and have transformed toxicology to a data-rich discipline. The increase in algorithm and computational power (Big Computer) now allow making Big Sense from evidence retrieval to its integration to predictions and the support to their reporting.

Toxicology is poised for substantial transformation *via* new technologies, especially AI, improving predictive toxicology, risk assessment, and mechanistic understanding. AI holds vast potential benefits and challenges for toxicology (Figure 1).

AI systems like machine learning algorithms can analyze large, complex datasets to identify patterns and relationships. In toxicology, these models can predict toxicity of new compounds based on existing data, replacing some animal testing. Deep learning shows particular promise for predictive toxicology by finding complex patterns using neural networks. This could improve prediction accuracy compared to simple machine learning models.

However, realizing these benefits presents significant challenges. High-quality, unbiased, and representative training data is essential for reliable predictions but requires substantial effort to develop toxicological databases meeting these criteria. While reducing animal studies, AI efficiency could also enable more substances to be assessed, raising new ethical questions. Interpretability poses another key challenge. Despite performance, deep learning models are poorly understood “black boxes,” limiting mechanistic insights - critical in toxicology.

In conclusion, AI presents exciting opportunities to enhance prediction, reduce animal testing, and otherwise advance toxicology. However, challenges related to data, ethics, and interpretability must be addressed through cross-disciplinary collaborative efforts to effectively integrate AI into toxicology in a manner ultimately promoting public and environmental health. Critically, AI cannot overcome inherent limitations of our methods and data - it does not absolve the need to improve the evidence generation process.

## Author contributions

TH: Writing – original draft, Writing – review & editing.
